# Truth and Error in Vaccination

**Published:** 1892-11

**Authors:** Stuart Close

**Affiliations:** Brooklyn, N. Y.


### INTERNATIONAL HAHNEMANNIAN ASSOCIATION MEETING OF 1892.

### TRUTH AND ERROR IN VACCINATION.

Stuart Close, M. D., Brooklyn, N. Y.

No one can read the history of vaccination without being deeply moved.

We are interested, at first, in the life and character of Jenner, its discoverer.

His originality, his energy, enthusiasm, perseverance, and courage, displayed during a long period of misunderstanding, ridicule and opposition, command our admiration, and characterize him as a man who must be accorded a high place among the physicians of his time.

His followers, in promulgating the new doctrine, have not fallen short of the standard thus set for them, and in the face of tremendous opposition have displayed qualities which have won for them a large measure of success.

Their zeal in gathering statistics and facts, and the ingenuity with which these are marshaled in support of their position are quite praiseworthy.

The constant, unrelenting pursuit of their object, the very audacity of which claims our admiration, all combine to make the study of this question a most fascinating one. Their object was, at first, to legalize compulsory vaccination, and when that was accomplished, to make it universal.

The ambition and dream of the vaccinationist, like Alexander, was "Universal Dominion."

But as we come to a study of vaccination itself, different feelings arise.

As we learn its origin, with all the repulsive details of the source of the virus, the method of its propagation and preparation, and realize that this vile product of an animal disease is introduced by inoculation directly into the human system, we are filled with disgust.

When, in the further pursuit of our study, we learn the effects of this operation ; that in many cases it is fatal, in other cases working serious and irreparable harm, and probably in all cases doing some harm (the amount depending upon the strength and resisting power of the subject), horror and astonishment take possession of us. We wonder at the extent to which such an operation, with such results, has gained acceptance and is practiced.

We are not surprised that it has excited opposition, and that men have rebelled against it, not only as a dangerous and repulsive operation, but, when it has been made compulsory, as an outrageous invasion of man's most sacred personal rights.

It is a curious fact that vaccination stands as an almost solitary example of legislative interference with a man's right to maintain the integrity of his own body.

We are reminded of the ancient Jewish circumcision as an example similar in some respects. Circumcision, however, had a religious significance, and as it not only was done by direct command of Jehovah, but had hygienic considerations in its favor, and entailed no possibility of disease or injury, it could be submitted to with good grace.

Circumcision was not compulsory in the same sense that vaccination is. There were penalties attached to non-compliance, but they were of a negative character only. The man who would not submit was not compelled to pay a fine, was not thrown into prison, nor deprived of his personal liberty in any way. He was simply debarred from being one of the "chosen people."

The records of the cases of some who have refused to submit to vaccination are not pleasant reading. Individuals have resisted, and have been arrested, brutally treated, fined, and thrown into prison.

Men have banded together to sustain each other in their defense of themselves and their families against the attack of the public vaccinator.

They have even exiled themselves from their native land in hopes of escaping the hated operation, only to be met at the gates of the new land by the official vaccinator, who compelled them to submit or be returned whence they came.

Great societies have been formed to fight the enemy in Parliament and in legislative halls, and they are still fighting. The London Society for the Abolition of Compulsory Vaccination is a large and powerful body, numbering in its ranks some of the greatest minds in England or of the age, and commanding the influence and sympathy of such men as Herbert Spencer, T. H. Huxley, and, in his lifetime, our own Hering.

The stupendous blunder out of which vaccination developed would have been ludicrous, if it had not been fraught with such terrible consequences. The brilliant Lady Mary Wortley Montagu, while traveling in the Orient, in 1721, learned that the Turks combatted small-pox by subcutaneous inoculation with the small-pox virus. She investigated the subject, became an enthusiastic convert, and, in her patriotic zeal, determined to introduce the custom into England. This was done, and the new operation soon became popular. It did not occur to the noble lady or her followers, however, that they were really introducing small-pox, for although most of the inoculated cases ran a mild course to a favorable termination, every one, of course, became a point of contagion, the same as an ordinary case of small-pox. The disease was thus spread rapidly, until it became a serious question as to how its ravages were to be stopped.

In 1798, Jenner invented vaccination, and introduced it as a substitute for inoculation.

From that day to this it has been the subject of endless controversy and strife. It has had its ardent supporters and strenuous opponents, each usually going to extremes in their positions and claims. As is usually the case, neither will admit any point of the others' contention.

I shall attempt to show that there is truth on both sides, and seek for some point at which these conflicting statements and positions may be harmonized.

Emerson exhorts us in one of his poems to "take the wheat and leave the chaff."

The philosopher and seeker after truth must often take this position of a winnower of chaff.

Diligent sifting will bring to light many good grains of wheat in every measure of chaff. The wildest and most absurd theory may contain one or more elements of truth, and be therefore worthy of the wise man's consideration. He will take the wheat and leave the chaff. He will listen patiently and humbly to what a smaller man would impatiently dismiss with a contemptuous sneer, and he will be rewarded. He will avoid a contentious or partisan attitude, and will not, therefore, always be found in sects, or subscribing to sharply-drawn creeds.

He will see "sermons in stones, books in the running brooks, and good in everything." He will be largely tolerant of all men and all systems, because he seeks always the truth, and knows that truth is very great, and is not easily to be compassed.

No sect is so narrow that does not possess some element of truth; no sect or system so broad as to include all truth.

Making large allowance for prejudice and for the partisan spirit which often makes extravagant claims and juggles with statistics, the truth appears to be that vaccination, to a limited extent, affords protection against small-pox.

The protection is not absolute in all cases. It is absolute in some cases. It is partial in some cases. It affords no protection in some cases.

It is here regarded only as a prophylactic measure.

According to its advocates, it may and does become a therapeutic measure, under certain circumstances, viz.:

"If vaccination is performed at any time within three days of exposure to small-pox, the vaccination will cause a certain degree of modification of the small-pox, and consequent protection" (R. H. M. S.).

To this we may yield a qualified assent.

On the other hand, it appears to be true, as its opponents claim, that vaccination is a repulsive and dangerous operation; that it introduces into the subject a virulent animal poison, the product of a loathsome disease in an animal; that this sets up a morbid process that may of itself either prove fatal or entail a lifetime of suffering and disease, or may so change and weaken the subjects' resisting power that they easily succumb to other diseases.

We do not jeopardize our standing as consistent Hahnemannians, or compromise our principles in any way by such admissions. We are rather following the example of Hahnemann, who not only admitted that cures were performed by his allopathic opponents, but sought out such cases, accounted for and explained them, and used them as illustrations of the truth of his newly-discovered therapeutic law.

He showed that the cure was wrought because the patient had, by chance, received the homœopathic simillimum, and he showed how this knowledge could be made available in all cases.

He was willing to be taught even by his enemies.

Pursuing a similar course in the study of the vaccination question, we shall be able to explain the reasons for both the good and the bad results attributed to it.

We have only to take it out of the field of polemics, and apply to it the same principles and the same course of reasoning that we use in discussing any other prophylactic or therapeutic remedy.

From ordinary vaccination has been obtained some good and some bad results.

That harm should be done is only to be expected from the very crude and unscientific method pursued. It cannot be otherwise. It is the same in the administration of any and all drugs.

When crude drugs are given in large doses, some cures will be performed, but great harm will be done in many other cases.

Even the brilliancy of the cures is too often dimmed by the complication with drug symptoms and the violent reactions excited. These are truisms with us.

That good results should be obtained in some cases is not surprising when we pursue this course a little further. It is easily explainable on purely homœopathic grounds.

*Vaccination owes any degree of efficiency it may possess, either as a prophylactic, or as a therapeutic measure, to its homœopathicity to the case in which it is employed, and the extent of the protection afforded, or of modification secured, is proportioned to its degree of similarity.*

This is a proposition which may be intelligently discussed, in the light of the principles of this Association.

Is vaccination homœopathic to small-pox? In other words, are the symptoms produced by vaccination similar to the symptoms of small-pox?

The simple statement of the question is all that is needed in this presence, and I need not take time to make or read a comparative table of symptoms. We have only to turn to the last volume of Hering's *Guiding Symptoms*, now so happily in our possession, and study the article on Vaccininum and Variolinum. The similarity of the two trains of symptoms has often been noted in a general way.

In the course of the vaccine disease we have, as it were, a case of small-pox in miniature, even to the development of the characteristic papule, vesicle, pustule, scab, and cicatrix.

There are those—and they are not all in the homœopathic ranks, either—who hold that the two diseases are identical in nature. Hering, in the *Guiding Symptoms*, combines the symptoms of Variol. and Vaccin. together in one proving. Here, then, is the explanation of the power of vaccination to protect some from infection, and to mitigate the symptoms of others suffering from small-pox.

The question of Posology now meets us here as in all cases. It is a fundamental question upon which rests tremendous issues.

If vaccination is a homœopathic procedure, and it is desirable or necessary to perform it, is the traditional and customary method the best one?

Shall we use the crude and deadly original virus, inserting it directly into the circulation by the lancet or scarifier, as is commonly done, or shall we use a homœopathic high potency of the virus, prepared and administered as are all our remedies?

Let us get the light of a kindred and closely-allied question upon it.

In a case requiring the administration of Psorinum, which the symptoms demand as the simillimum, shall we use the original crude pus from the sore of a diseased negro, introducing it directly into the circulation, or shall we use a high potency?

In a case requiring Medorrhinum, shall we inject into the circulation a little of the crude gonorrhœal virus, or shall we give the 45M or CM potency?

Are not the cases alike? What member of this Association hesitates a moment to answer, "Give the potency, of course!" And that is precisely the right thing to do in all cases, including vaccination.

What havoc should we produce if we used Psorinum, Medorrhinum, Syphilinum, Lachesis, Belladonna, or any other poisonous drug in the same manner as Vaccininum is used! What wrecked lives; what suffering and death; what outraged feelings; what suits for malpractice; what "lamp-post receptions" we should have! And yet the people submit to vaccination with all its evils for the most part uncomplainingly because they have been blinded and they are ignorant of a better way.

My contention is that as it is unnecessary, and even criminal, to use Psorinum, Medorrhinum, Syphilinum, Lachesis, Belladonna, or any other poisonous drug in a crude form and introduced directly into the circulation, so it is unncessary to use vaccine virus in that way. To vaccinate in the ordinary way seems to me a direct and flagrant violation of the fundamental principles of Homœopathy and of this Association.

In the administration of medicines for the prevention of disease we must be governed by the same law and the same principles as in the treatment and cure of disease. This is a logical necessity. Hahnemann clearly taught this, notably when he recommended Belladonna as a preventive of scarlet fever, and directed that it be given in the thirtieth potency.

If we use the potentiated remedy for treatment we must also use it for prevention if we are to secure the same superior results.

If it is not necessary in the treatment of a case of small-pox to inject subcutaneously the crude virus of Vaccininum, Variolinum, *or any other drug*, then it is not necessary to use that method in the attempt to protect against small-pox.

This seems to me to be the only tenable position for a Hahnemannian.

We are called upon to provide a means of protection against small-pox, and ordinary vaccination is forbidden us by its very nature.

The potentiated medicine overcomes the difficulty and solves the problem, as it has solved all kindred problems.

Hahnemann's chief glory lies in his discovery of potentiation. This is peculiarly his own discovery, and it constitutes his chief claim to scientific immortality and the gratitude of mankind.

Others before him had seen more or less clearly the other truths he promulgated, but to him alone was revealed the grand and far-reaching truth of potentiation.

It was this discovery which enabled him to overcome the difficulties which had always stood in the way of the general practical application of the law of the similars.

The similar remedy given in large doses and in crude form only aggravated the disease or killed the patient. The potentiated remedy cured. Jenner, too, made a great, though a partial, discovery. The view I have taken of this question accords to him his true position in the ranks of the world's benefactors. Jenner's discovery is not complete until it is brought into its proper relation to Hahnemann's law of Posology. Then it assumes its proper place in our armory of weapons against disease, and Jenner takes his true place as *the discoverer of a new remedy*, a nosode to be sure, but valuable when proved in combatting certain forms of disease, among others small-pox.

Jenner did not know how properly to apply his remedy in order to secure the best results. The method he adopted is merely a clumsy imitation of the Turk's inoculation.

If he had taken counsel of Hahnemann, as he might have done, he might have learned a better way, and the world to-day have been better off and healthier. As it is, vaccination has probably done more harm than good.

In *Homœopathic Vaccination*, therefore, we have an agent for good worthy of our acceptance. Here is truth undefiled by error, good wheat freed from chaff. Turning to our literature we find the records of many cases of small-pox treated with Variolinum and Vaccininum in high potency, and always with good results. Indeed, the results in some cases so treated are marvelous, the disease running its entire course in about a week, with very little inconvenience to the patient, and no pitting. Dr. Fincke has, I believe, collected a number of such cases. From the use of no other remedies have such results been obtained as the records of these cases show. We are justified in claiming at least, then, that as a method of treatment in small-pox, using the higher potencies of Variolinum or Vaccininum is far superior to ordinary vaccination.

In the light of our experience with other drugs as prophylactics, are we not also justified in claiming that what I have called "Homœopathic Vaccination" is equally superior to ordinary vaccination as a preventive of small-pox? And may we not hope that this may become the practice, at least, of all Hahnemannians?

